# Impact of Transcatheter Mitral and Tricuspid Valve Repair on Hepatic Function and Outcomes in Patients with Cirrhosis or Advanced Liver Disease—A Personalized Approach

**DOI:** 10.3390/jcm15051883

**Published:** 2026-03-01

**Authors:** Tina Bečić, Ivana Jukić, Petra Šimac Prižmić, Ivona Matulić, Hana Đogaš, Mislav Radić, Josipa Radić, Jonatan Vuković, Damir Fabijanić

**Affiliations:** 1 Department of Cardiovascular Diseases, University Hospital of Split, 21000 Split, Croatia; tina.becic@gmail.com (T.B.); damirfabijanic62@gmail.com (D.F.); 2 Department of Internal Medicine, Division of Gastroenterology, University Hospital of Split, 21000 Split, Croatia; 3 Faculty of Health Sciences, University of Split, 21000 Split, Croatia; 4 Department of Internal Medicine, Division of Rheumatology, Allergology and Clinical Immunology, University Hospital of Split, 21000 Split, Croatia; petra_simac@hotmail.com (P.Š.P.); mislavradic@gmail.com (M.R.); 5 Private Clinic Matulic, Osjecka Ulica 24a, 21000 Split, Croatia; ivonamatulic@yahoo.com; 6 Department of Neurology, University Hospital of Split, 21000 Split, Croatia; hana.dogas@gmail.com; 7 Department of Internal Medicine, School of Medicine, University of Split, 21000 Split, Croatia; josiparadic1973@gmail.com; 8 Department of Internal Medicine, Division of Nephrology, Dialysis and Arterial Hypertension, University Hospital of Split, 21000 Split, Croatia; 9 Department of Clinical Propedeutics, School of Medicine, University of Split, 21000 Split, Croatia

**Keywords:** transcatheter edge-to-edge repair, mitral regurgitation, tricuspid regurgitation, chronic liver disease, cardiohepatic syndrome, MELD score, MELD-XI, hepatic dysfunction, personalized medicine

## Abstract

**Background:** Transcatheter edge-to-edge repair (TEER) has emerged as an established treatment option for patients with severe mitral (MR) and tricuspid regurgitation (TR) who are at high surgical risk. Patients referred for TEER frequently present with advanced comorbidities, including cirrhosis or chronic liver disease (CLD). Hepatic dysfunction, driven by chronic venous congestion and impaired cardiac output, represents a key yet underrecognized determinant of prognosis in this population. The impact of TEER on hepatic function and outcomes in patients with advanced liver disease remains incompletely defined. **Methods:** This systematic review was conducted in accordance with PRISMA 2020 guidelines and registered in PROSPERO. A comprehensive literature search of PubMed, Scopus, Web of Science, and the Cochrane Library was performed up to 16 January 2026, without language restrictions. Studies evaluating mitral or tricuspid TEER in adult patients with cirrhosis, chronic or advanced liver disease, congestive hepatopathy, or cardiohepatic syndrome were included. Hepatic function was assessed using biochemical markers, clinical diagnoses, or composite scores such as Model for End-Stage Liver Disease (MELD) score and Model for End-Stage Liver Disease Excluding INR (MELD-XI). A qualitative synthesis was performed due to heterogeneity in study design and outcome reporting. **Results:** Twelve studies were included, comprising prospective and retrospective cohorts, registry-based analyses, mechanistic studies, and one illustrative case report. Six studies evaluated mitral TEER (M-TEER) and six tricuspid (T-TEER). Across both valve interventions, impaired baseline hepatic function was consistently associated with increased mortality and adverse clinical outcomes. MELD and MELD-XI scores emerged as robust prognostic markers following both M-TEER and T-TEER. Successful reduction in valvular regurgitation was associated with stabilization or improvement of hepatic parameters in selected patients, particularly after T-TEER. However, advanced cardiohepatic syndrome and limited hepatic reserve were linked to poor outcomes despite procedural success. **Conclusions:** Hepatic dysfunction is a powerful determinant of prognosis in patients undergoing M-TEER and T-TEER. While TEER may improve hepatic congestion and liver-related parameters in selected patients, outcomes are highly dependent on baseline hepatic reserve and global hemodynamic status. A personalized approach integrating hepatic assessment into patient selection and risk stratification is essential to optimize outcomes in this complex and growing population.

## 1. Introduction

Transcatheter edge-to-edge repair (TEER) has become an established therapeutic option for patients with severe mitral (MR) and tricuspid regurgitation (TR) who are considered at high or prohibitive surgical risk. Since the landmark randomized trials demonstrated the feasibility, safety, and clinical efficacy of transcatheter mitral valve repair (M-TEER) compared with surgery or optimal medical therapy [1,2,3,4], TEER has rapidly evolved from an alternative strategy into a cornerstone of contemporary structural heart disease management. Long-term follow-up data have further confirmed durable reductions in regurgitation severity and improved clinical outcomes in selected patient populations [5]. Parallel to technological refinement and expanding operator experience, the clinical profile of patients referred for TEER has substantially changed. In real-world practice, candidates for transcatheter mitral or tricuspid valve repair are increasingly characterized by advanced age, multiple comorbidities, and complex extracardiac organ involvement [6,7,8]. Among these, hepatic dysfunction represents a particularly underappreciated yet clinically meaningful determinant of procedural risk and long-term prognosis. Heart failure (HF) and significant atrioventricular valve regurgitation exert profound effects on hepatic physiology through chronic venous congestion, reduced forward cardiac output, and neurohormonal activation, giving rise to congestive hepatopathy and the broader spectrum of cardiohepatic syndrome [9,10]. Progressive liver dysfunction in this setting is associated with worsening functional status, increased hospitalization rates, and excess mortality [11,12]. Importantly, hepatic impairment may remain clinically silent until advanced stages, limiting its recognition in routine preprocedural risk stratification. Traditional surgical risk scores inadequately capture the prognostic impact of liver dysfunction. Consequently, liver-specific scoring systems such as the Model for End-Stage Liver Disease (MELD) original score and its modified variant excluding the international normalized ratio (MELD-XI) have been increasingly adopted in cardiovascular populations [13,14,15]. These scores have demonstrated prognostic value in patients with advanced HF, those undergoing cardiac surgery, and more recently, in patients treated with transcatheter valve interventions. Emerging evidence suggests that hepatic dysfunction is not merely a bystander but a powerful predictor of outcomes following TEER. Observational studies and registry data have consistently shown that impaired liver function, assessed by MELD or MELD-XI scores, is associated with increased mortality and adverse clinical events after both M-TEER and T-TEER [16,17,18,19,20]. Conversely, successful reduction in regurgitation severity may lead to partial reversibility of congestive hepatopathy, supporting the concept that “fixing the valve may heal the liver” in selected patients [21]. Patients with established cirrhosis or advanced chronic liver disease (CLD) represent an especially vulnerable subgroup. Historically, these patients have been underrepresented in randomized trials and are often excluded from surgical interventions due to prohibitively high perioperative risk [22,23]. Preliminary data indicate that TEER may offer a less invasive therapeutic alternative in this population; however, outcomes appear highly heterogeneous and strongly influenced by baseline hepatic reserve [24]. This heterogeneity underscores the limitations of a “one-size-fits-all” approach and highlights the need for individualized patient assessment. In this context, a personalized approach integrating hepatic function into patient selection, procedural planning, and post-interventional risk stratification becomes essential. Understanding which patients with liver dysfunction derive meaningful clinical benefit from TEER, and which remain at high risk despite successful valve repair, represents a critical unmet clinical need. Therefore, this systematic review aims to evaluate the impact of transcatheter mitral and tricuspid valve repair on hepatic function and clinical outcomes in patients with cirrhosis or advanced liver disease, with a specific focus on the prognostic role of baseline hepatic dysfunction. By integrating available evidence across different hepatic risk phenotypes, we seek to inform personalized patient selection and risk stratification in this high-risk population.

## 2. Materials and Methods

This systematic review was conducted in accordance with the Preferred Reporting Items for Systematic Reviews and Meta-Analyses (PRISMA) 2020 guidelines [25]. The completed PRISMA 2020 checklist is included in the Supplementary Materials. The study protocol was prospectively registered in the International Prospective Register of Systematic Reviews (PROSPERO ID: CRD420261288144). The methodological framework was designed to capture the heterogeneity of hepatic phenotypes, assessment strategies, and outcome reporting in patients undergoing M-TEER and T-TEER, in line with the personalized approach outlined in the Introduction.

### 2.1. Study Design and Data Sources

A comprehensive literature search was performed in PubMed, Scopus, Web of Science and Cochrane Library. The search aimed to identify studies evaluating the impact of M-TEER and/or T-TEER on hepatic function and clinical outcomes in patients with cirrhosis, CLD, advanced hepatic dysfunction, congestive hepatopathy, or cardiohepatic syndrome. The final search was conducted on 16 January 2026. No language restrictions were applied, and studies published in languages other than English were considered eligible if sufficient data could be extracted.

The electronic search strategy was based on the following combination of keywords and Boolean operators and was applied consistently across all databases: (“transcatheter edge-to-edge repair” OR TEER OR MitraClip OR TriClip OR “transcatheter mitral valve repair” OR “transcatheter tricuspid valve repair” OR “mitral TEER” OR “tricuspid TEER”) AND (“mitral regurgitation” OR “tricuspid regurgitation”) AND (“cirrhosis” OR “chronic liver disease” OR “advanced liver disease” OR “hepatic dysfunction” OR “liver dysfunction” OR “congestive hepatopathy” OR “cardiohepatic syndrome”) AND (“MELD” OR “MELD-XI” OR “hepatic function” OR bilirubin OR albumin OR “liver enzymes” OR prognosis OR outcomes OR mortality). To enhance transparency and reproducibility, the complete electronic search strategies for each database, including all Boolean operators and applied filters, are provided in the Supplementary Materials (Supplementary S1). Database-specific yields and search dates are summarized in Supplementary Table S1. A detailed list of full-text exclusions with reasons is provided in Supplementary Table S2.

### 2.2. Eligibility Criteria

Studies were considered eligible if they included adult patients undergoing M-TEER and/or T-TEER and if cirrhosis, CLD, advanced hepatic dysfunction, congestive hepatopathy, or cardiohepatic syndrome was documented, or if hepatic function was objectively assessed. Given the heterogeneity across studies, cirrhosis and advanced liver disease were defined according to each study’s operational criteria, including clinical diagnosis, imaging findings, laboratory thresholds, or administrative coding (ICD-based definitions). Cardiohepatic syndrome was defined as the coexistence of cardiac dysfunction and biochemical or clinical evidence of hepatic impairment, as operationalized within individual studies.

Eligible studies were required to report hepatic parameters—such as bilirubin, albumin, liver enzymes, or MELD- or MELD-XI-based scores—and/or clinical outcomes in relation to hepatic status. Original research articles were included, encompassing prospective and retrospective cohort studies, registry-based analyses, mechanistic investigations, as well as selected case reports.

Case reports were included exclusively in the qualitative synthesis when they provided mechanistic insight or illustrative evidence of potentially reversible congestive hepatopathy following transcatheter edge-to-edge repair and were excluded from any quantitative or comparative analyses. The case report was included exclusively for illustrative and mechanistic purposes and did not contribute to comparative or prognostic conclusions. Reviews, editorials, conference abstracts without available full-text data, and non-human studies were excluded.

### 2.3. Operational Definitions of Hepatic Phenotypes

To enhance consistency across heterogeneous study definitions, key hepatic phenotypes were categorized using predefined operational criteria. Advanced chronic liver disease (CLD)/cirrhosis was defined as a documented clinical diagnosis of cirrhosis or advanced liver disease, presence of portal hypertension, or explicit classification as cirrhotic in the original study. Congestive hepatopathy was defined as liver dysfunction attributed primarily to chronic venous congestion in the setting of heart failure or severe valvular regurgitation, in the absence of primary intrinsic liver disease. Cardiohepatic syndrome (CHS) was defined as the coexistence of cardiac dysfunction and hepatic impairment, as described in the respective study, or as a combined phenotype characterized by abnormal hepatic biomarkers (e.g., bilirubin, albumin, liver enzymes, or MELD-based scores) in the context of heart failure or significant atrioventricular valve disease. Where studies used alternative terminology or composite definitions, phenotypes were mapped to these predefined categories for structured synthesis.

### 2.4. Study Selection and Data Extraction

**Figure 1:**
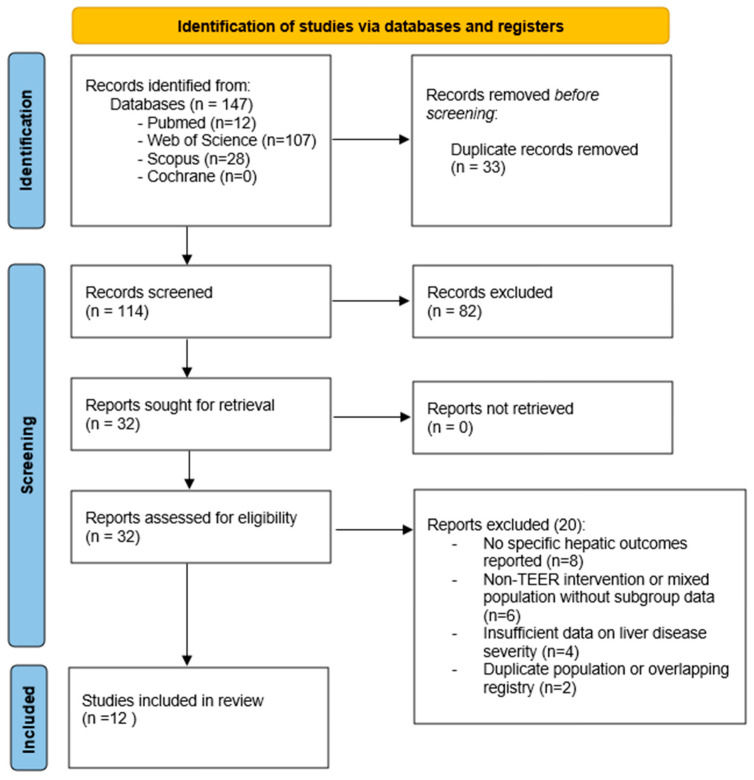
PRISMA 2020 flow diagram of study identification, screening, eligibility assessment, and inclusion in the systematic review.

After removal of duplicates, titles and abstracts were independently screened by two reviewers (TB and PŠP). Full-text articles were subsequently assessed for eligibility. Disagreements were resolved by consensus and, when necessary, consultation with a third reviewer. The study selection process is summarized in the PRISMA 2020 flow diagram (Figure 1).

Data extraction was performed using a standardized data collection form, including study design, patient population, valve pathology and type of TEER (mitral or tricuspid), methods of hepatic assessment, and outcomes relevant to liver function and prognosis. The main characteristics of the included studies are summarized in Table 1.

### 2.5. Risk of Bias Assessment

**Figure 2:**
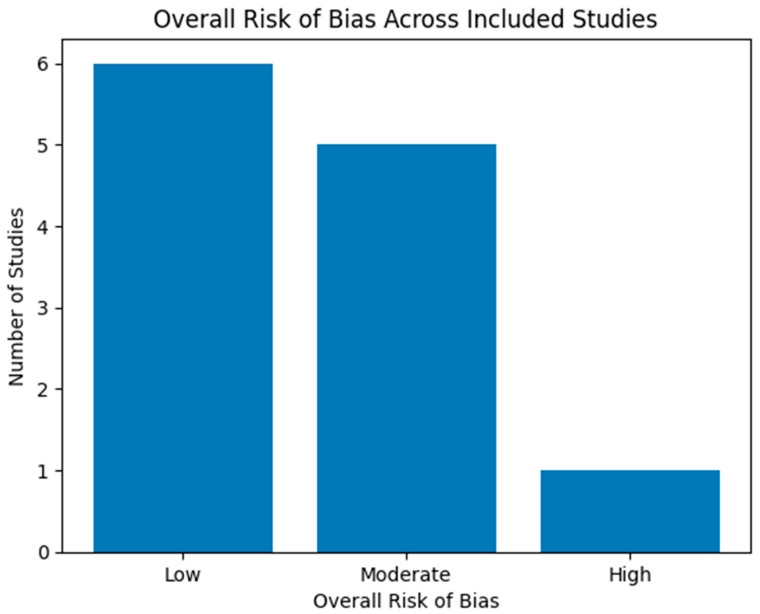
Overall risk of bias across included studies. This figure depicts the overall risk of bias among the included studies. Most studies demonstrated a low to moderate risk of bias, consistent with their observational design. One case report was classified as high risk by design and was included for illustrative purposes only.

Risk of bias was independently assessed by two reviewers (TB and PŠP) using the Risk Of Bias In Non-randomized Studies of Interventions (ROBINS-I) tool, which is specifically designed for observational studies. The following domains were evaluated: bias due to confounding, selection of participants, classification of interventions, deviations from intended interventions, missing data, measurement of outcomes, and selective reporting. Based on domain-level judgments, an overall risk of bias was assigned to each included study. The results of the risk of bias assessment are summarized in Table 2, while a graphical overview of the risk of bias across studies is presented in Figure 2. The single case report included in the review was considered to be at high risk of bias by design and was therefore not assessed using the ROBINS-I tool. Instead, it was evaluated descriptively and included exclusively for illustrative and mechanistic purposes.

### 2.6. Data Synthesis

**Figure 3:**
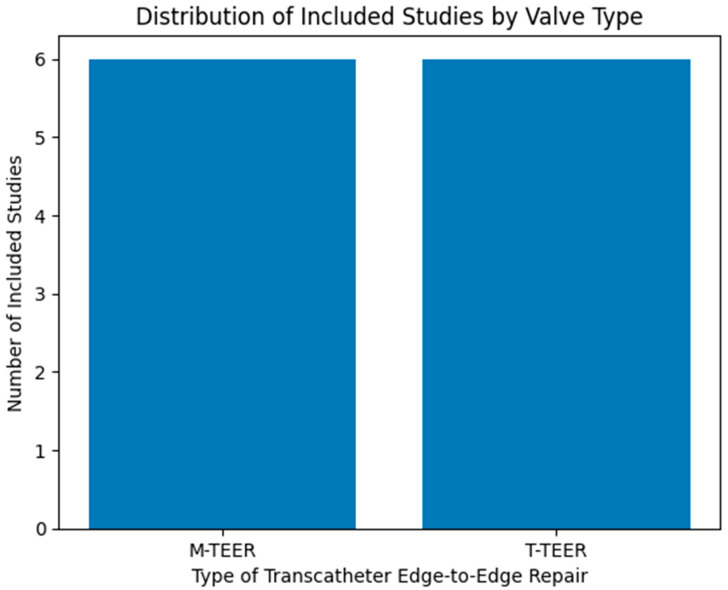
Distribution of included studies by transcatheter edge-to-edge repair type. This figure illustrates the distribution of the included studies according to the type of transcatheter edge-to-edge repair. An equal number of studies evaluated mitral (M-TEER) and tricuspid (T-TEER) interventions, reflecting a balanced representation of both valve pathologies within the systematic review. This balanced distribution supports a comprehensive assessment of cardiohepatic interactions across different valvular phenotypes.

For each included study, we extracted sample size, duration of follow-up, primary outcome timepoints, adjusted effect estimates (hazard ratios or odds ratios with 95% confidence intervals where reported), and key variables included in multivariable adjustment models. Given the heterogeneity of study designs, hepatic assessment methods, and outcome reporting, a qualitative synthesis was performed. Particular emphasis was placed on the relationship between baseline hepatic function, post-procedural changes in liver-related parameters, and clinical outcomes following TEER. Where applicable, findings were stratified by valve type (M-TEER versus T-TEER) to reflect differences in hemodynamic burden and cardiohepatic interaction. The distribution of included studies according to TEER type is illustrated in Figure 3. To account for variability in multivariable risk adjustment across observational studies, we systematically extracted information on covariates included in adjusted models. Particular attention was given to key hemodynamic confounders, including right ventricular function, cardiac output, pulmonary pressures, renal function, and age. A structured sensitivity synthesis was performed, restricting interpretation to studies that adjusted for at least one major hemodynamic parameter, in order to assess the robustness of the association between hepatic dysfunction and outcomes. In addition to hepatic biomarkers and clinical outcomes, we systematically extracted available data on invasive and non-invasive hemodynamic parameters, including right atrial pressure, central venous pressure, cardiac output, pulmonary artery pressures, and right ventricular function. Particular emphasis was placed on studies reporting correlations between hemodynamic measures and changes in hepatic parameters following TEER.

## 3. Results

### 3.1. Study Selection

The systematic search identified a total number of records across PubMed, Scopus, Web of Science, and the Cochrane Library. After removal of duplicates, titles and abstracts were screened for relevance. Full-text articles were subsequently assessed for eligibility, resulting in the inclusion of twelve studies in the qualitative synthesis. The study selection process is detailed in the PRISMA 2020 flow diagram (Figure 1). Among the included studies, six evaluated outcomes following M-TEER, and six focused on T-TEER, reflecting a balanced representation of both valve pathologies (Figure 3). In total, 147 records were identified across all databases, including 12 from PubMed, 28 from Scopus, 107 from Web of Science, and 0 from the Cochrane Library. After removal of 33 duplicates, 114 records underwent title and abstract screening. Of these, 82 records were excluded. Thirty-two full-text articles were assessed for eligibility, of which 20 were excluded for predefined reasons, including absence of hepatic assessment, non-TEER interventions, insufficient outcome reporting, or ineligible study design. A detailed breakdown of database-specific yields, search dates, complete electronic search strategies, and full-text exclusions with reasons is provided in the Supplementary Materials.

### 3.2. Characteristics of Included Studies

The main characteristics of the included studies are summarized in Table 1. Study designs comprised prospective and retrospective cohort studies, registry-based analyses, and mechanistic investigations. One case report was included for qualitative and illustrative purposes only. The majority of studies were observational in nature and derived from high-volume tertiary centers or large administrative databases. Patient populations were predominantly elderly and characterized by advanced valvular disease and multiple comorbidities. Hepatic function was assessed using a variety of parameters, including biochemical markers (bilirubin, albumin, liver enzymes), clinical diagnoses of CLD or cirrhosis, and composite scores such as MELD score or MELD-XI.

### 3.3. Mitral Transcatheter Edge-to-Edge Repair and Hepatic Outcomes

Six studies evaluated hepatic function and outcomes in patients undergoing M-TEER. Impaired baseline hepatic function was consistently associated with adverse outcomes. In a retrospective cohort study, Spieker (2019) demonstrated that higher MELD-XI scores were independently associated with increased mortality following M-TEER [17]. Similarly, registry-based analyses by Khan (2021) and Sawalha (2021) showed that patients with CLD undergoing M-TEER experienced higher in-hospital risk compared with patients without liver disease, although in-hospital outcomes were acceptable relative to the high baseline surgical risk of this population; however, long-term prognosis remained influenced by hepatic reserve [24,27]. Prospective data further highlighted the clinical relevance of cardiohepatic interactions. Stolz (2023) identified cardiohepatic syndrome, defined by combined cardiac and hepatic dysfunction, as a strong predictor of adverse outcomes following M-TEER [26]. In a large multicenter registry, Yeo (2025) reported that hepatic dysfunction, reflected by abnormal liver enzymes and bilirubin levels, was associated with increased early mortality after M-TEER [28]. One case report described successful M-TEER in a liver transplant candidate, with subsequent improvements in congestive hepatopathy, illustrating the potential reversibility of hepatic dysfunction in selected patients [29].

### 3.4. Tricuspid Transcatheter Edge-to-Edge Repair and Hepatic Outcomes

Six studies focused on hepatic function and outcomes in patients undergoing T-TEER. Across these studies, baseline hepatic impairment was a strong determinant of prognosis. In a prospective cohort study, Karam (2019) demonstrated significant improvements in bilirubin levels and MELD-XI scores following successful T-TEER, suggesting a beneficial impact on hepatic congestion [16]. However, several studies emphasized that the presence of advanced cardiohepatic syndrome portends a worse prognosis. Stolz (2022) showed that patients with cardiohepatic syndrome undergoing T-TEER had significantly higher mortality rates [19]. Tanaka (2021) similarly reported that elevated MELD-XI scores were associated with increased mortality following T-TEER [18]. Mechanistic and phenotyping studies provided additional insight into the cardiohepatic interaction. Unterhuber (2021) demonstrated that outcomes after T-TEER differed according to cardiac output phenotype, highlighting the complex relationship between systemic hemodynamics and liver function [30]. Rommel (2023) further linked venous congestion and stressed blood volume to hepatic dysfunction in patients with severe TR [31]. Registry data from Torres (2022) confirmed that T-TEER is feasible in patients with significant comorbidity burden, including liver disease, with acceptable in-hospital outcomes relative to baseline risk; however, longer-term prognosis remained dependent on hepatic reserves [32].

### 3.5. Risk of Bias Across Studies

The overall risk of bias assessment is summarized in Table 2 and Figure 2. Most cohort and registry-based studies were judged to have low to moderate risk of bias, consistent with their observational design. The single case report was classified as high risk of bias by design and was included exclusively for qualitative illustration.

### 3.6. Summary of Findings

Across both M-TEER and T-TEER, impaired hepatic function—assessed by biochemical markers, clinical diagnoses, or composite scores such as MELD score and MELD-XI—was consistently associated with worse clinical outcomes. Conversely, successful reduction in valvular regurgitation was associated with stabilization or improvement of hepatic parameters in selected patients, particularly following T-TEER. When findings were stratified according to predefined hepatic phenotype categories (cirrhosis/advanced CLD, congestive hepatopathy, or cardiohepatic syndrome), the association between impaired hepatic function and adverse outcomes remained directionally consistent across subgroups, although the magnitude of risk varied according to baseline hepatic reserve and hemodynamic severity. Adjusted effect estimates and key multivariable covariates are detailed in Table 1 and Supplementary Table S6.

### 3.7. Hemodynamic–Hepatic Interactions

Several included studies provided detailed hemodynamic characterization of patients undergoing TEER, particularly in tricuspid cohorts. Elevated right atrial pressure, impaired cardiac output, and markers of systemic venous congestion were consistently associated with worse hepatic function and adverse outcomes. Mechanistic investigations demonstrated that reductions in venous congestion following successful T-TEER were paralleled by improvements in bilirubin levels and MELD-XI scores in selected patients. These findings support the concept that hepatic dysfunction in this population is closely linked to hemodynamic derangement rather than representing isolated intrinsic liver disease.

### 3.8. Sensitivity Synthesis According to Risk Adjustment

The extent of multivariable adjustment varied across studies. Several prospective cohorts and registry analyses adjusted for key hemodynamic variables, including RV function, cardiac output phenotype, pulmonary pressures, and renal function, whereas administrative database studies were limited to demographic and comorbidity-based adjustments. When findings were restricted to studies incorporating hemodynamic adjustment, impaired hepatic function remained independently associated with adverse outcomes, supporting the robustness of the observed prognostic relationship.

## 4. Discussion

### 4.1. Principal Findings and Clinical Context

In this systematic review, we synthesized available evidence on the impact of transcatheter mitral and tricuspid valve repair on hepatic function and clinical outcomes in patients with cirrhosis or advanced liver disease. The principal finding of this systematic review is that hepatic dysfunction—whether defined biochemically, clinically, or by composite scoring systems such as MELD score or MELD-XI—represents a consistent and powerful determinant of prognosis following transcatheter edge-to-edge repair. These findings are consistent with the broader body of evidence establishing TEER as an effective treatment for severe atrioventricular valve regurgitation in high-risk populations, as demonstrated in randomized trials and long-term follow-up studies for mitral regurgitation [1,2,3,4,5,33] and, more recently, for tricuspid regurgitation [34,35,36,37]. However, these landmark trials largely underrepresented patients with advanced extracardiac organ dysfunction, particularly liver disease, underscoring the importance of focused analyses such as current review scores such as the MELD score or MELD-XI, which are consistent and powerful determinants of prognosis following TEER. Across both mitral and tricuspid interventions, impaired baseline liver function was associated with increased mortality and adverse outcomes, whereas successful reduction in valvular regurgitation was associated with stabilization or improvement of hepatic parameters in selected patients.

### 4.2. Cardiohepatic Interactions as a Determinant of TEER Outcomes

The association between HF, valvular regurgitation, and hepatic dysfunction is well established. Chronic venous congestion, reduced forward cardiac output, and systemic inflammation contribute to the development of congestive hepatopathy and the broader cardiohepatic syndrome [9,10,12,38]. In heart failure populations, biomarkers of liver dysfunction have emerged as powerful prognostic indicators, often outperforming traditional cardiac risk markers [11]. Within the TEER population, these pathophysiological interactions are particularly pronounced. Across the studies included in this review, hepatic dysfunction consistently reflected not only comorbidity burden but also the severity and chronicity of underlying hemodynamic derangement. Mechanistic investigations further support this concept, linking venous congestion, stressed blood volume, and impaired hepatic perfusion to adverse outcomes, particularly in patients with severe tricuspid regurgitation [30,31,39]. Collectively, these data reinforce the concept that liver dysfunction in TEER candidates functions both as a marker and a mediator of advanced disease.

### 4.3. Prognostic Role of MELD and MELD-XI Scores

Composite liver scores, particularly MELD score and MELD-XI, emerged as robust prognostic tools across multiple studies. Originally developed for survival prediction in end-stage liver disease [13,40], these scores have been successfully adapted to cardiovascular populations, including advanced HF and mechanical circulatory support candidates [14,15]. In the context of TEER, both mitral and tricuspid cohorts demonstrated a strong association between elevated MELD or MELD-XI scores and mortality [16,17,18,19,20]. Importantly, MELD-XI offers practical advantages in anticoagulated patients, a frequent scenario in TEER candidates. These findings support the incorporation of liver-specific scoring systems into preprocedural risk stratification and reinforce the need for a personalized approach beyond conventional surgical risk scores.

### 4.4. Mitral TEER in Patients with Liver Disease

Evidence regarding M-TEER in patients with CLD remains limited but informative. Registry-based analyses demonstrated that M-TEER is feasible in patients with cirrhosis or CLD, with lower in-hospital mortality compared with surgical repair [24,27]. Nevertheless, hepatic dysfunction remained a strong predictor of adverse outcomes, consistent with findings from real-world MitraClip registries [6,7,8,41]. Prospective data further highlighted the relevance of cardiohepatic syndrome as a distinct clinical entity associated with worse outcomes after M-TEER [26]. The illustrative case of a liver transplant candidate undergoing successful M-TEER underscores the potential reversibility of congestive hepatopathy in carefully selected patients [29], supporting the concept that valve repair may serve as a bridge or adjunct to advanced hepatologic therapies.

### 4.5. Tricuspid TEER and Hepatic Function: A Bidirectional Relationship

TR represents a paradigmatic model of cardiohepatic interaction. The included studies consistently demonstrated that T-TEER can lead to improvements in hepatic parameters, particularly in patients with reversible congestion [16,18]. However, advanced cardiohepatic syndrome was associated with poor prognosis despite procedural success [19,20]. Large registries and randomized trials confirmed the feasibility and safety of T-TEER in high-risk populations [32,36,37,42], while mechanistic studies emphasized the importance of right ventricular function, cardiac output phenotype, and venous congestion in determining outcomes [30,31,43]. These data collectively suggest that hepatic dysfunction should be interpreted in the context of global hemodynamics rather than as an isolated comorbidity.

### 4.6. Implications for Patient Selection and Personalized Decision-Making

The heterogeneity observed across studies underscores the limitations of a one-size-fits-all approach. Current ESC/EACTS and ACC expert consensus documents increasingly emphasize comprehensive patient assessment, including extracardiac organ function, in the management of valvular heart disease [44,45,46]. In patients with liver disease, multidisciplinary evaluation involving cardiology, hepatology, and cardiac surgery is essential. Our findings support a personalized framework in which hepatic function is integrated into patient selection, timing of intervention, and post-procedural management. In selected patients, TEER may interrupt the vicious cycle of congestion and hepatic dysfunction—an idea encapsulated by the concept of “fix the heart, heal the liver” [21]. Conversely, advanced cirrhosis with limited hepatic reserve may attenuate the benefits of valve intervention, as suggested by surgical and interventional outcomes in cirrhotic populations [22,23,47].

### 4.7. Clinical and Research Implications

From a clinical perspective, routine assessment of hepatic function—including MELD-XI scoring and congestion-related biomarkers—should be considered in all candidates for transcatheter edge-to-edge repair. Future research should focus on refining hepatic phenotyping, defining thresholds of reversibility and incorporating liver-related endpoints into TEER trials. Future prospective studies should incorporate standardized pre- and post-procedural hemodynamic assessment, including right heart catheterization or validated non-invasive surrogates of venous congestion and cardiac output. Such an approach may allow more precise identification of patients with reversible congestive hepatopathy and better delineation of the cardiohepatic trajectory following intervention. Prospective studies and registries with standardized hepatic assessment will be essential, particularly as the landscape of transcatheter tricuspid interventions, including valve replacement strategies, continues to expand [48,49,50,51]. Importantly, hepatic dysfunction in patients undergoing transcatheter valve interventions must be interpreted within a broader hemodynamic and systemic context. Pulmonary hypertension, right ventricular–pulmonary artery coupling and biventricular interactions are key determinants of outcomes after both mitral and tricuspid TEER and directly modulate venous congestion and hepatic perfusion [52,53]. Registry and multicenter data further underscore the prognostic relevance of residual or progressive tricuspid regurgitation following M-TEER, highlighting the interdependence of atrioventricular valve disease and systemic congestion [54,55]. Despite advances in device technology and procedural success with newer-generation mitral and tricuspid systems [56,57,58,59], extracardiac organ dysfunction, particularly liver disease, remains a major source of outcome heterogeneity. Hepatic manifestations range from subclinical biochemical abnormalities to overt congestive hepatopathy or cirrhosis and may include disproportionate cholestatic patterns in heart failure settings [60]. In patients with established cirrhosis, cardiovascular interventions require careful consideration of bleeding risk, thrombosis, and altered pharmacokinetics [61,62]. Collectively, these observations emphasize that outcomes after M-TEER and T-TEER cannot be adequately understood through a valve-centric lens alone. Rather, they reflect the complex interplay between cardiac mechanics, pulmonary vascular disease, systemic congestion, and hepatic reserve, underscoring the need for a multidisciplinary and integrative framework to optimize patient selection and timing of intervention. Emerging advances in non-invasive liver assessment, including biomarker-based fibrosis panels and imaging modalities such as elastography, may further enhance risk stratification in patients with suspected or established cirrhosis. Recent comprehensive reviews on innovative diagnostic and therapeutic strategies in liver cirrhosis underscore the importance of integrating biochemical, imaging, and systemic markers to refine prognostic evaluation and guide personalized management strategies. Such approaches may be particularly relevant in TEER candidates, in whom distinguishing reversible congestive hepatopathy from advanced intrinsic cirrhosis remains clinically challenging [63].

### 4.8. Clinical Impact in Patients with Cirrhosis: Beyond Laboratory Parameters

While composite liver scores such as MELD and MELD-XI provide valuable prognostic information, their clinical relevance in patients with cirrhosis extends beyond laboratory changes alone. In this population, systemic venous congestion and elevated right-sided pressures contribute not only to biochemical abnormalities but also to clinically meaningful manifestations, including ascites, peripheral edema, fatigue, reduced exercise tolerance, and impaired quality of life.

Reduction in tricuspid or mitral regurgitation through TEER may alleviate systemic congestion, potentially improving ascite control and functional class in selected patients. Although formal quality-of-life and decompensation endpoints were inconsistently reported across included studies, mechanistic data suggest that lowering right atrial pressure and improving forward cardiac output may reduce the risk of hepatic decompensation events, such as worsening ascites or hepatic encephalopathy, particularly in patients with reversible congestive hepatopathy.

Importantly, the intersection between valvular heart disease and cirrhosis has implications for transplant candidacy and timing. In carefully selected individuals, TEER may serve as a bridge strategy by stabilizing cardiohepatic status, reducing venous congestion, and potentially improving transplant eligibility. Conversely, advanced intrinsic cirrhosis with limited hepatic reserve may not derive the same degree of clinical benefit despite successful valve intervention. As transcatheter mitral and tricuspid therapies continue to evolve in complexity and scope, careful multidisciplinary evaluation remains essential to balance procedural risk, hepatic reserve, and long-term patient-centered outcomes. As highlighted in recent state-of-the-art reviews of transcatheter mitral and tricuspid interventions, patient selection, procedural complexity, and systemic comorbidity burden remain central determinants of outcome [64,65].

### 4.9. Limitations

The overall body of evidence is predominantly observational, and several sources of bias warrant careful consideration when interpreting our findings. Selection bias may have occurred in single-center cohorts, where patients with advanced hepatic decompensation were less likely to be referred for or undergo TEER, potentially underestimating the true prognostic impact of severe liver dysfunction. In contrast, registry-based and administrative studies may be affected by misclassification bias, particularly when hepatic disease was identified through diagnostic coding rather than standardized clinical assessment, which may not capture disease severity or distinguish between congestive hepatopathy and intrinsic cirrhosis, thereby potentially attenuating observed associations. Residual confounding represents another important limitation. Hepatic dysfunction frequently coexists with advanced right ventricular failure, pulmonary hypertension, and impaired cardiac output—conditions that independently influence prognosis. Although several studies adjusted for major hemodynamic parameters, variability in adjustment strategies, and the limited granularity of administrative and registry-based datasets may have influenced the magnitude of effect estimates, leading to partial overestimation or attenuation of the independent prognostic impact of hepatic dysfunction. Nevertheless, the consistency of directional associations across studies with varying degrees of bias and adjustment supports a clinically meaningful relationship rather than a purely confounded signal. The generalizability of our findings may be limited by the predominance of single-center cohorts and registry-based analyses derived from high-volume tertiary referral centers. Such settings may not fully reflect broader clinical practice, particularly in regions with different referral pathways, patient selection thresholds, or access to transcatheter therapies. Future multicenter registries and prospective studies across diverse healthcare systems are needed to externally validate these findings. Particular emphasis should be placed on the systematic inclusion and stratification of patients with established cirrhosis and advanced chronic liver disease, who remain underrepresented in contemporary TEER trials.

This review has additional limitations. The included studies were heterogeneous in hepatic assessment methods and outcome definitions, which precluded a quantitative meta-analysis and limits direct comparability across reports. The absence of randomized data specifically addressing patients with advanced liver disease limits causal inference regarding the impact of TEER on hepatic function and downstream clinical outcomes. Additionally, one case report was included for illustrative purposes only and does not contribute to comparative conclusions, but was retained to provide mechanistic insight into potentially reversible congestive hepatopathy following successful intervention.

## 5. Conclusions

Hepatic dysfunction represents a powerful and independent determinant of prognosis in patients undergoing transcatheter edge-to-edge repair of the mitral and tricuspid valves and constitutes a central component of the cardiohepatic syndrome in this population. Accumulating evidence indicates that impaired liver function reflects not only comorbidity burden but also the severity and chronicity of underlying hemodynamic derangement, particularly venous congestion and right-sided heart failure. While successful transcatheter valve repair may result in stabilization or partial improvement of hepatic function in selected patients, clinical outcomes remain highly heterogeneous and are strongly influenced by baseline hepatic reserve, the presence of advanced chronic liver disease, and global cardiopulmonary hemodynamics. These findings suggest the existence of a critical window during which intervention may favorably modify the cardiohepatic trajectory, whereas advanced or irreversible liver dysfunction may limit the potential benefit of TEER despite procedural success. Accordingly, a personalized and integrative approach that incorporates systematic assessment of hepatic function alongside comprehensive hemodynamic and cardiopulmonary evaluation is essential to optimize patient selection, procedural timing, and post-interventional risk stratification. As transcatheter therapies continue to expand across broader risk spectra and increasingly complex patient populations, future studies should prioritize standardized hepatic phenotyping and liver-related endpoints to refine decision-making and improve outcomes in patients with advanced extracardiac comorbidity.

## Figures and Tables

**Table 1:** Characteristics of Included Studies.

First Author (Year)	Study Design	Valve/Procedure	Sample Size (n)	Population	Hepatic Assessment	Follow-Up	Primary Outcome(s)	Adjusted Effect Estimates (95% CI)	Key Covariates in Multivariable Models
Spieker (2019) [17]	Retrospective cohort	M-TEER	133	Severe MR undergoing MitraClip	MELD-XI	Median 458 days	All-cause mortality	HR per MELD-XI increase: 1.08 (95% CI 1.03–1.14)	Age, sex, LVEF, NYHA class, renal function
Stolz (2023) [26]	Prospective cohort	M-TEER	249	MR with/without cardiohepatic syndrome	CHS definition, bilirubin, albumin	12 months	Mortality, HF hospitalization	CHS vs. no CHS: HR 2.12 (95% CI 1.29–3.49)	Age, sex, LVEF, RV function, renal function
Khan (2021) [27]	Registry analysis (NIS)	M-TEER	15,270	MR with vs. without CLD	Administrative CLD codes	In-hospital	In-hospital mortality, complications	Adjusted OR for mortality: 1.29 (95% CI 1.05–1.58)	Age, sex, comorbidities, renal failure
Sawalha (2021) [24]	Registry comparison (NIS)	M-TEER vs. surgery	875 (CLD subgroup)	MR with cirrhosis/CLD	Administrative CLD codes	In-hospital	In-hospital mortality	TEER vs. surgery: OR 0.46 (95% CI 0.28–0.74)	Age, sex, comorbidities
Yeo (2025) [28]	Multicenter registry	M-TEER	2381	MR undergoing TEER	Bilirubin, liver enzymes	30 days	Early mortality	Abnormal liver tests: OR 1.67 (95% CI 1.21–2.31)	Age, LVEF, renal function, frailty
Ates (2022) [29]	Case report	M-TEER	1	Liver transplant candidate	Clinical cirrhosis	6 months	Hepatic recovery	Not applicable	Not applicable
Karam (2019) [16]	Prospective cohort	T-TEER	126	Severe TR	Bilirubin, MELD-XI	6 months	Mortality, liver function change	MELD-XI per point: HR 1.09 (95% CI 1.02–1.17)	Age, renal function, RV function
Stolz (2022) [19]	Prospective cohort	T-TEER	170	Severe TR	Cardiohepatic syndrome	Median 1.1 years	All-cause mortality	CHS vs. no CHS: HR 2.37 (95% CI 1.39–4.04)	Age, RV function, CO, renal function
Unterhuber (2021) [30]	Phenotype cohort	T-TEER	160	Functional TR	Hemodynamic–hepatic interaction	12 months	Mortality	Low CO phenotype: HR 2.10 (95% CI 1.20–3.69)	CO, RV function, PAP
Tanaka (2021) [18]	Retrospective cohort	T-TEER	145	Severe TR	MELD-XI	Median 400 days	All-cause mortality	MELD-XI ≥15: HR 2.03 (95% CI 1.16–3.56)	Age, renal function, LVEF
Rommel (2023) [31]	Mechanistic cohort	T-TEER	70	Severe TR	Congestive biomarkers	6 months	Hemodynamic–hepatic link	Not reported	Hemodynamics-focused
Torres (2022) [32]	Registry analysis (NIS)	T-TEER	1410	TR with comorbidities	Administrative liver codes	In-hospital	In-hospital mortality	Adjusted OR: NS	Demographics, comorbidities

Abbreviations: CHS, cardiohepatic syndrome; CLD, chronic liver disease; CO, cardiac output; MELD score, Model for End-Stage Liver Disease; MELD-XI, Model for End-Stage Liver Disease excluding international normalized ratio; MR, mitral regurgitation; M-TEER, mitral transcatheter edge-to-edge repair; NIS, National Inpatient Sample; TEER, transcatheter edge-to-edge repair; TR, tricuspid regurgitation; T-TEER, tricuspid transcatheter edge-to-edge repair.

**Table 2:** Risk of Bias Assessment of Included Studies.

Study (First Author, Year)	Study Design	Selection Bias	Performance Bias	Detection Bias	Attrition Bias	Reporting Bias	Overall Risk of Bias
Spieker, 2019 [17]	Retrospective cohort	Moderate	Low	Low	Low	Low	Moderate
Stolz, 2023 (M-TEER) [26]	Prospective cohort	Low	Low	Low	Low	Low	Low
Khan, 2021 [27]	Registry (NIS)	Moderate	Low	Moderate	Low	Low	Moderate
Sawalha, 2021 [24]	Registry comparison	Moderate	Low	Moderate	Low	Low	Moderate
Yeo, 2025 [28]	Multicenter registry	Moderate	Low	Low	Low	Low	Moderate
Ates, 2022 [29]	Case report	High	Low	Low	Low	Low	High
Karam, 2019 [16]	Prospective cohort	Low	Low	Low	Low	Low	Low
Stolz, 2022 (T-TEER) [19]	Prospective cohort	Low	Low	Low	Low	Low	Low
Unterhuber, 2021 [30]	Phenotype cohort	Low	Low	Low	Low	Low	Low
Tanaka, 2021 [18]	Retrospective cohort	Moderate	Low	Low	Low	Low	Moderate
Rommel, 2023 [31]	Mechanistic cohort	Low	Low	Low	Low	Low	Low
Torres, 2022 [32]	Registry (NIS)	Moderate	Low	Moderate	Low	Low	Moderate

Abbreviations: NIS, National Inpatient Sample; M-TEER, mitral transcatheter edge-to-edge repair; T-TEER, tricuspid transcatheter edge-to-edge repair.

## Data Availability

No new data were created or analyzed in this study. Data sharing is not applicable to this article.

## Supplementary Materials

The following supporting information can be downloaded at https://www.mdpi.com/article/10.3390/jcm15051883/s1: Supplementary S1. Complete electronic search strategy used across all databases. Supplementary Table S1. Database-specific search dates and yields. Supplementary Table S2. Full-text exclusions with reasons. Supplementary Table S3. Hemodynamic–hepatic interactions in studies evaluating transcatheter edge-to-edge repair. Supplementary Table S4. Adjusted effect estimates and key multivariable covariates reported in included studies. Supplementary S5. PRISMA 2020 Checklist.

## Abbreviations

CLD	Chronic Liver Disease
HF	Heart Failure
MELD	Model for End-Stage Liver Disease
MELD-XI	Model for End-Stage Liver Disease Excluding INR
MR	Mitral Regurgitation
M-TEER	Mitral Transcatheter Edge-to-Edge Repair
PRISMA	Preferred Reporting Items for Systematic Reviews and Meta-Analyses
PROSPERO	International Prospective Register of Systematic Reviews
ROBINS-I	Risk Of Bias In Non-randomized Studies of Interventions
TEER	Transcatheter Edge-to-Edge Repair
TR	Tricuspid Regurgitation
T-TEER	Tricuspid Transcatheter Edge-to-Edge Repair
