# Supplementary material for: Impact of Transcatheter Mitral and Tricuspid Valve Repair on Hepatic Function and Outcomes in Patients with Cirrhosis or Advanced Liver Disease—A Personalized Approach
Source: J Clin Med. 2026 Mar 1;15(5):1883. doi: 10.3390/jcm15051883 (PMC12986291; doi:10.3390/jcm15051883)
Supplement: Supplementary file 1 [file jcm-15-01883-s001.zip › jcm-4156329-supplementary S1-S4.pdf]

Supplementary S1

Complete electronic search strategy.

The electronic search strategy was based on a predefined combination of keywords and Boolean operators and was applied consistently across all databases.

The following search string was used: ("transcatheter edge-to-edge repair" OR TEER OR MitraClip OR TriClip OR "transcatheter mitral valve repair" OR "transcatheter tricuspid valve repair" OR "mitral TEER" OR "tricuspid TEER") AND ("mitral regurgitation" OR "tricuspid regurgitation") AND ("cirrhosis" OR "chronic liver disease" OR "advanced liver disease" OR "hepatic dysfunction" OR "liver dysfunction" OR "congestive hepatopathy" OR "cardiohepatic syndrome") AND ("MELD" OR "MELD-XI" OR "hepatic function" OR bilirubin OR albumin OR "liver enzymes" OR prognosis OR outcomes OR mortality).

Supplementary Table S1

Database-specific search dates and yields.

Database	Search date	Records identified (n)
PubMed	16 January 2026	12
Scopus	16 January 2026	28
Web of Science	16 January 2026	107
Cochrane Library	16 January 2026	0
Total	-	147

Search strategies were executed without language restrictions. Duplicate records were removed prior to screening.

Supplementary Table S2

Full-text exclusions with reasons (n = 20).

Study (first author, year)	Full reference	Reason for exclusion
Baldus et al., 2012	**Baldus, S.; Schillinger, W.; Franzen, O.; Bekeredjian, R.; Sievert, H.; Schofer, J.; Kuck, K.-H.; Konorza, T.; Möllmann, H.; Hehrlein, C.; et al. MitraClip Therapy in Daily Clinical Practice: Initial Results from the German Transcatheter Mitral Valve Interventions (TRAMI) Registry.** *European Journal of Heart Failure*, 2012; **14**(9): 1050–1055. https://doi.org/10.1093/eurjhf/hfs079	No specific hepatic outcomes reported
Puls et al., 2016	**Puls, M.; Lubos, E.; Boekstegers, P.; von Bardeleben, R.S.; Ouarrak, T.; Butter, C.; Zuern, C.S.; Bekeredjian, R.; Sievert, H.; Nickenig, G.; et al. One-Year Outcomes and Predictors of Mortality after MitraClip Therapy in Contemporary Clinical Practice: Results from the German Transcatheter Mitral Valve Interventions (TRAMI) Registry.** *European Heart Journal*, 2016; **37**(8): 703–712. **https://doi.org/10.1093/eurheartj/ehv627**	No specific hepatic outcomes reported
Sorajja et al., 2017	**Sorajja, P.; Vemulapalli, S.; Feldman, T.; Mack, M.; Holmes, D.R., Jr.; Stebbins, A.; Kar, S.; Thourani, V.; Ailawadi, G. Outcomes with Transcatheter Mitral Valve Repair in the United States: An STS/ACC TVT Registry Report.** *Journal of the American College of Cardiology*, 2017; **70**(19): 2315–2327. https://doi.org/10.1016/j.jacc.2017.09.015	No specific hepatic outcomes reported
Maisano et al., 2013	**Maisano, F.; Franzen, O.; Baldus, S.; Schäfer, U.; Hausleiter, J.; Butter, C.; Ussia, G.P.; Sievert, H.; Richardt, G.; Widder, J.D.; et al. Percutaneous Mitral Valve Interventions in the Real World: Early and 1-Year Results from the ACCESS-EU, a Prospective, Multicenter, Nonrandomized Post-Approval Study of the MitraClip Therapy in Europe.** *Journal of the American College of Cardiology*, 2013; **62**(12): 1052–1061. https://doi.org/10.1016/j.jacc.2013.02.094	No specific hepatic outcomes reported

Ailawadi et al., 2019	**Ailawadi, G.; Lim, D.S.; Mack, M.J.; Trento, A.; Kar, S.; Grayburn, P.A.; Glower, D.D.; Wang, A.; Foster, E.; Qasim, A.; et al. One-Year Outcomes After MitraClip for Functional Mitral Regurgitation.** *Circulation*, 2019; **139**(1): 37–47. https://doi.org/10.1161/CIRCULATIONAHA.117.031733	No specific hepatic outcomes reported
von Bardeleben et al., 2023	**von Bardeleben, R.S.; Mahoney, P.; Morse, M.A.; Price, M.J.; Denti, P.; Maisano, F.; Rogers, J.H.; Rinaldi, M.; De Marco, F.; Rollefson, W.; et al. 1-Year Outcomes with Fourth-Generation Mitral Valve Transcatheter Edge-to-Edge Repair from the EXPAND G4 Study.** *JACC: Cardiovascular Interventions*, 2023; **16**(23): 2600–2610. https://doi.org/10.1016/j.jcin.2023.09.029	No specific hepatic outcomes reported
Nickenig et al., 2017	**Nickenig, G.; Kowalski, M.; Hausleiter, J.; Braun, D.; Schofer, J.; Yzeiraj, E.; Rudolph, V.; Friedrichs, K.; Maisano, F.; Taramasso, M.; et al. Transcatheter Treatment of Severe Tricuspid Regurgitation with the Edge-to-Edge MitraClip Technique.** *Circulation*, 2017; **135**(19): 1802–1814. https://doi.org/10.1161/CIRCULATIONAHA.116.024848	No specific hepatic outcomes reported
Lurz et al., 2021	**Lurz, P.; von Bardeleben, R.S.; Weber, M.; Sitges, M.; Sorajja, P.; Hausleiter, J.; Denti, P.; Trochu, J.-N.; Nabauer, M.; Tang, G.H.L.; et al. Transcatheter Edge-to-Edge Repair for Treatment of Tricuspid Regurgitation.** *Journal of the American College of Cardiology*, 2021; **77**(3): 229–239. https://doi.org/10.1016/j.jacc.2020.11.038	No specific hepatic outcomes reported
Lopez-Delgado et al., 2015	**Lopez-Delgado, J.C.; Esteve, F.; Javierre, C.; Ventura, J.L.; Mañez, R.; Farrero, E.; Torrado, H.; Rodríguez-Castro, D.; Carrio, M.L. Influence of Cirrhosis in Cardiac Surgery Outcomes.** *World Journal of Hepatology*, 2015; **7**(5): 753–760. https://doi.org/10.4254/wjh.v7.i5.753	Non-TEER intervention or mixed population without subgroup data

Garatti et al., 2021	**Garatti, A.; Daprati, A.; Cottini, M.; Russo, C.F.; Dalla Tomba, M.; Troise, G.; Salsano, A.; Santini, F.; Scrofani, R.; Nicolò, F.; et al. Cardiac Surgery in Patients with Liver Cirrhosis (CASTER) Study: Early and Long-Term Outcomes.** *The Annals of Thoracic Surgery*, 2021; **111**(4): 1242–1251. https://doi.org/10.1016/j.athoracsur.2020.06.110	Non-TEER intervention or mixed population without subgroup data
Hewing et al., 2021	**Hewing, B.; Mattig, I.; Knebel, F.; Stangl, V.; Laule, M.; Stangl, K.; Dreger, H. Renal and Hepatic Function of Patients with Severe Tricuspid Regurgitation Undergoing Inferior Caval Valve Implantation.** *Scientific Reports*, 2021; **11**: 21800. https://doi.org/10.1038/s41598-021-01322-2	Non-TEER intervention or mixed population without subgroup data
Li et al., 2025	**Li, Z.; Lin, D.; Miao, J.; Fan, J.; Lu, F.; Zhang, X.; Pan, W.; Zhou, D.; Ge, J. Effects of Transcatheter Tricuspid Valve Replacement on Hepatic and Renal Function in Severe Tricuspid Regurgitation.** *International Journal of Cardiology: Heart & Vasculature*, 2025; **59**: 101714. https://doi.org/10.1016/j.ijcha.2025.101714	Non-TEER intervention or mixed population without subgroup data
Pétursson et al., 2023	**Pétursson, I.; Amabile, A.; Degife, E.; Morrison, A.; Waldron, C.; Bin Mahmood, S.U.; Ragnarsson, S.; Krane, M.; Geirsson, A. Outcomes of Patients with Advanced Liver Disease Undergoing Cardiac Surgery.** *JTCVS Open*, 2023; **16**: 532–539. https://doi.org/10.1016/j.xjon.2023.07.001	Non-TEER intervention or mixed population without subgroup data
Malinchoc et al., 2000	**Malinchoc, M.; Kamath, P.S.; Gordon, F.D.; Peine, C.J.; Rank, J.; ter Borg, P.C. A Model to Predict Poor Survival in Patients Undergoing Transjugular Intrahepatic Portosystemic Shunts.** *Hepatology*, 2000; **31**(4): 864–871. https://doi.org/10.1053/he.2000.5852	Non-TEER intervention or mixed population without subgroup data

Torres et al., 2022	**Torres, C.; Lozier, M.R.; Davidson, C.J.; Ailawadi, G.; Donatelle, M.; Vedantam, K.; Kodali, S.K.; Leon, M.B.; Beohar, N. In-Hospital Outcomes of Transcatheter Tricuspid Valve Repair: An Analysis from the National Inpatient Sample.** *Journal of the Society for Cardiovascular Angiography & Interventions*, 2022; **1**: 100414. https://doi.org/10.1016/j.jscai.2022.100414	Insufficient data on liver disease severity
Nguyen et al., 2025	**Nguyen, A.; Khan, M.Z.; Alruwaili, W.; Nassar, S.; Khan, Z.; Thomas, P.; Elhosseiny, S.; Siordia, J.; Kovach, R.; Raza, M. Inpatient Outcomes of Tricuspid Transcatheter Edge-to-Edge Repair in the United States Based on Sex.** *Journal of the Society for Cardiovascular Angiography & Interventions*, 2025; **4**: 102644. https://doi.org/10.1016/j.jscai.2025.102644	Insufficient data on liver disease severity
Wu et al., 2022	**Wu, J.F.; Jha, P.; Doucette, S.; Abid, H. Significantly Elevated Alkaline Phosphatase Caused by Congestive Hepatopathy in the Setting of Heart Failure with Preserved Ejection Fraction.** *WMJ (Wisconsin Medical Journal)*, 2022; **121**(2): E14–E17.<br>**PMID:** 35442588	Insufficient data on liver disease severity
Brankovic et al., 2023	**Brankovic, M.; Lee, P.; Pyrsopoulos, N.; Klapholz, M. Cardiac Syndromes in Liver Disease: A Clinical Conundrum.** *Journal of Clinical and Translational Hepatology*, 2023; **11**(4): 975–986.<br>https://doi.org/10.14218/JCTH.2022.00294	Insufficient data on liver disease severity
Mauri et al., 2013	**Mauri, L.; Foster, E.; Glower, D.D.; Apruzzese, P.; Massaro, J.M.; Herrmann, H.C.; Hermiller, J.; Gray, W.; Wang, A.; Pedersen, W.R.; et al. 4-Year Results of a Randomized Controlled Trial of Percutaneous Repair versus Surgery for Mitral Regurgitation.** *Journal of the American College of Cardiology*, 2013; **62**(4): 317–328.<br>https://doi.org/10.1016/j.jacc.2013.04.030	Duplicate population / overlapping registry

Feldman et al., 2015	**Feldman, T.; Kar, S.; Elmariah, S.; Smart, S.C.; Trento, A.; Siegel, R.J.; Apruzzese, P.; Fail, P.; Rinaldi, M.J.; Smalling, R.W.; et al. Randomized Comparison of Percutaneous Repair and Surgery for Mitral Regurgitation: 5-Year Results of EVEREST II.** *Journal of the American College of Cardiology*, 2015; **66**(25): 2844–2854. https://doi.org/10.1016/j.jacc.2015.10.018	Duplicate population / overlapping registry

Supplementary Table S3. Hemodynamic–Hepatic Interactions in Studies Evaluating Transcatheter Edge-to-Edge Repair

First Author (Year)	Valve / TEER Type	Study Design	Key Hemodynamic Parameters Assessed	Hepatic Parameters Assessed	Main Hemodynamic–Hepatic Findings	Clinical Implications
**Karam (2019)** [16]	T-TEER	Prospective cohort	Right atrial pressure, venous congestion surrogates	Bilirubin, MELD-XI	Reduction in venous congestion after successful T-TEER was associated with significant improvement in bilirubin levels and MELD-XI score	Suggests partial reversibility of congestive hepatopathy following TR reduction
**Stolz (2022)** [19]	T-TEER	Prospective cohort	RV function, systemic congestion	Cardiohepatic syndrome definition	Presence of cardiohepatic syndrome reflected advanced hemodynamic derangement and was associated with increased mortality	Advanced CHS identifies patients with limited hepatic reversibility
**Unterhuber (2021)** [30]	T-TEER	Phenotype cohort	Cardiac output phenotype, RV function	MELD-XI, liver biomarkers	Low cardiac output phenotype was associated with worse hepatic function and poorer outcomes despite TEER	Highlights importance of forward flow in hepatic recovery
**Rommel (2023)** [31]	T-TEER	Mechanistic cohort	Stressed blood volume, venous congestion	Congestion-related hepatic biomarkers	Increased venous congestion and stressed blood volume correlated with hepatic dysfunction	Confirms venous congestion as a key driver of liver impairment
**Tanaka (2021)** [18]	T-TEER	Retrospective cohort	Indirect congestion markers	MELD-XI	Higher MELD-XI reflected combined hepatic and hemodynamic impairment and predicted mortality	MELD-XI integrates hepatic and circulatory risk
**Stolz (2023)** [26]	M-TEER	Prospective cohort	RV function, global hemodynamics	Bilirubin, albumin, CHS	Cardiohepatic syndrome represented a combined hemodynamic–hepatic phenotype associated with worse outcomes	Supports integrated cardiohepatic risk stratification
**Yeo (2025)** [28]	M-TEER	Multicenter registry	Not invasively assessed	Liver enzymes, bilirubin	Abnormal hepatic biomarkers likely reflected advanced systemic congestion and predicted early mortality	Liver tests act as surrogate markers of hemodynamic severity
**Ates (2022)** [29]	M-TEER	Case report	Clinical signs of congestion	Clinical cirrhosis	Successful M-TEER led to improvement in congestive hepatopathy in a transplant candidate	Illustrates potential reversibility in selected patients

Abbreviations: CHS, cardiohepatic syndrome; MELD, Model for End-Stage Liver Disease; MELD-XI, Model for End-Stage Liver Disease excluding INR; M-TEER, mitral transcatheter edge-to-edge repair; RV, right ventricle; TEER, transcatheter edge-to-edge repair; TR, tricuspid regurgitation; T-TEER, tricuspid transcatheter edge-to-edge repair

Supplementary Table S4. Adjusted effect estimates and key multivariable covariates

Study (year)	TEER type	Hepatic variable	Outcome	Adjusted effect estimate	Key covariates included
**Spieker et al., 2019**	M-TEER	MELD-XI (>16 vs lower)	1-year all-cause mortality	**HR 3.39** (95% CI 1.25–9.19), p = 0.016	Age, sex, NT-proBNP, hemoglobin, renal function, residual MR
**Tanaka et al., 2021**	M-TEER	MELD-XI (>11 vs ≤11)	Composite outcome (all-cause mortality or HF hospitalization at 2 years)	**HR 1.34** (95% CI 1.02–1.77), p = 0.04	Age, sex, LVEF, TR severity, renal function, MR aetiology
**Stolz et al., 2022**	T-TEER	Cardiohepatic syndrome (CHS)	1-year all-cause mortality	**HR 1.86** (95% CI 1.10–3.14), p < 0.05	Age, sex, renal function, RV function, TR severity, NYHA class
**Stolz et al., 2023**	M-TEER	Cardiohepatic syndrome (CHS type I/II)	2-year all-cause mortality	**HR 1.49** (95% CI 1.05–2.12), p = 0.027	Age, sex, LVEF, TAPSE, eGFR, NYHA class, residual MR
**Karam et al., 2019**	T-TEER	Baseline hepatic dysfunction (bilirubin / liver enzymes)	Clinical outcomes and survival at 6 months	**Adjusted association reported** (no single pooled HR)	Age, sex, renal function, NYHA class, TR severity, LVEF
